# Retraction Note: Targeting transforming growth factor beta (TGF-β) using Pirfenidone, a potential repurposing therapeutic strategy in colorectal cancer

**DOI:** 10.1038/s41598-025-34893-5

**Published:** 2026-01-19

**Authors:** Hamid Jamialahmadi, Seyedeh Elnaz Nazari, Hamid TanzadehPanah, Ehsan Saburi, Fereshteh Asgharzadeh, Fatemeh Khojasteh-Leylakoohi, Maryam Alaei, Mahdi Mirahmadi, Fatemeh Babaei, Seyedeh Zahra Asghari, Saeide Mansouri, Ghazaleh Khalili-Tanha, Mina Maftooh, Hamid Fiuji, Seyed Mahdi Hassanian, Gordon A. Ferns, Majid Khazaei, Amir Avan

**Affiliations:** 1 Metabolic Syndrome Research Center, Mashhad University of Medical Sciences, Mashhad, Iran; 2 Basic Sciences Research Institute, Mashhad University of Medical Sciences, Mashhad, Iran; 3 Medical Genetics Research Center, Mashhad University of Medical Sciences, Mashhad, Iran; 4 Antimicrobial Resistance Research Center, Mashhad University of Medical Sciences, Mashhad, Iran; 5 Department of Pharmacology, Faculty of Pharmacy, Mashhad University of Medical Sciences, Mashhad, Iran; 6 Division of Medical Education, Brighton and Sussex Medical School, Falmer, Brighton, BN1 9PH Sussex UK; 7 College of Medicine, University of Warith Al-Anbiyaa, Karbala, Iraq; 8 School of Mechanical, Medical and Process Engineering, Queensland University of Technology, 2 George Street, Brisbane, QLD 4000 Australia; 9 Faculty of Health, School of Biomedical Sciences, Queensland University of Technology, Brisbane, Australia

Retraction of: *Scientific Reports* 10.1038/s41598-023-41550-2, published online 01 September 2023

The Editors have retracted this Article.

After publication, concerns has been raised about the data and the inconsistencies between its presentation and the description in the methods section, as well as the conduct of the animal experiments. The Editors requested explanation and the raw data underlying the paper. Only some of the data was provided and did not contain metadata to allow verification of its veracity, nor address the main concerns. Specifically:

• Although the methods state that at least two independent experiments were performed in triplicate, this is not borne out by the data provided. This means that most results are based on the groups of 2, making the choice of ANOVA for the analysis inappropriate;

• Fig. 1e CT-26 5-FU Day 0 and Control Day 3 appear highly similar;

• Fig. 1e CT-26 5-FU + PFD Day 1 and Control Day 2 appear highly similar;

• In Figure 2b p-values are shown to be below 0.0001 for all comparisons for CT-26, and to follow the same threshold patters for comparisons for SW-480. for CT-26 error bars are also not shown. The Authors were not able to explain this, nor provide raw data for this panel;

• According to the measurements of the tumour sizes provided by the Authors for experiments shown in Figure 3, tumour sizes in some of the animals exceeded the size at which the animals should be sacrificed as early as at Day 15. However, the experiments were not terminated until Day 19;

• Some of the original data provided by the Authors was mislabelled (i.e. described as other data in the Article), while some does not match any data in the paper (e.g. Fig. 6b kidney 5-FU).

Overall, the Editors have lost the confidence in the integrity of the data and results presented in this Article.

Majid Khazaei disagrees with the retraction, Fereshteh Asgharzadeh and Hamid Jamialahmadi have not explicitly stated about agreement or disagreement to the retraction. Remaining Authors did not respond to the correspondence about this retraction.

